# Adherence to the Healthy Nordic Food Index is associated with reduced plasma levels of inflammatory markers in patients with heterozygous familial hypercholesterolemia

**DOI:** 10.1016/j.athplu.2024.10.003

**Published:** 2024-10-24

**Authors:** Eirin B. Løvheim, Kjetil Retterstøl, Ingunn Narverud, Martin P. Bogsrud, Bente Halvorsen, Thor Ueland, Pål Aukrust, Kirsten B. Holven

**Affiliations:** a Department of Nutrition, Institute of Basic Medical Sciences, University of Oslo, P.O Box 1046 Blindern, 0317, Oslo, Norway; b Lipid Clinic, Oslo University Hospital, Norway; c Norwegian National Advisory Unit on Familial Hypercholesterolemia, Oslo University Hospital, Norway; d Unit for Cardiac and Cardiovascular Genetics, Oslo University Hospital, Oslo, Norway; e Research Institute for Internal Medicine, Oslo University Hospital, Oslo, Norway; f Section of Clinical Immunology and Infectious Diseases, Oslo University Hospital, Rikshospitalet, Oslo, Norway; g Institute of Clinical Medicine, Faculty of Medicine, University of Oslo, Norway; h Thrombosis Research Center (TREC), Division of Internal Medicine, University Hospital of North Norway, Tromsø, Norway

**Keywords:** Familial hypercholesterolemia, Inflammation, Atherosclerosis, Diet, Healthy

## Abstract

**Background and aims:**

Familial hypercholesterolemia (FH) is an inherited disease associated with hypercholesterolemia, and dietary treatment is part of the treatment. We aimed to assess the dietary pattern in relation to the Healthy Nordic Food Index (HNFI) in adults with and without heterozygous FH (HeFH), and to examine the associations between dietary quality and biomarkers related to cardiovascular disease in adults with HeFH.

**Methods:**

We included 205 adults (≥18 years) with HeFH who received follow-up at the Lipid Clinic in Oslo and compared them to controls (n = 228). Dietary intake was assessed using a food frequency questionnaire and dietary quality was assessed using the HNFI. Blood samples were analysed for levels of blood lipids, plasma fatty acids (FAs), and markers of inflammation and platelet activation.

**Results:**

The HeFH patients (median 60 years; 50.2 % female; 25.9 % in secondary prevention) had lower intake of total and saturated fat compared to controls (32.6 energy percent (E%) vs. 34.9 E%, and 9.6 E% vs 12.0 E%, respectively; p < 0.001 for both). In the HeFH patients, increasing dietary quality was associated with increased plasma levels of the n-3 polyunsaturated FAs (PUFAs) eicosapentaenoic acid and docosahexaenoic acid, and the n-6 PUFA linoleic acid, and lower plasma levels of the inflammatory cytokines Tumor Necrosis Factor and interleukin-6, and of the platelet-derived inflammatory cytokines Platelet Factor 4 and Neutrophil-Activating Peptide-2.

**Conclusion:**

Norwegian patients with HeFH followed up at a Lipid Clinic eat healthier than controls. Adherence to a healthy dietary pattern is associated with higher plasma levels of n-3 and n-6 PUFA, and lower levels of inflammatory markers, including platelet markers. This may suggest that adherence to an overall healthy dietary pattern might be beneficial for HeFH patients independent of the cholesterol-lowering effect of the diet.

## Highlights

• Patients with HeFH and controls had similar adherence to the Healthy Nordic Food Index (HNFI).
• HeFH patients had lower intake of saturated fat, red and processed meats, dietary cholesterol, and alcohol than controls.
• HeFH patients had higher intake of protein, omega-3 fatty acids, vegetables, and dietary fibre than controls.
• In HeFH patients, increasing HNFI adherence was associated with higher plasma levels of n-3 and n-6 PUFAs.
• In HeFH patients, increasing HNFI adherence was associated with lower levels of inflammatory markers.

## Graphical abstract

The figure was partly generated using pictures from Servier Medical Art. Servier Medical Art by Servier is licensed under a Creative Commons Attribution 3.0 Unported License (https://creativecommons.org/licenses/by/3.0/).Abbreviations: HeFH, heterozygous familial hypercholesterolemia; FFQ, food frequency questionnaire; SFA, saturated fatty acids; FAs, fatty acids; HNFI, Healthy Nordic Food Index; EPA, eicosapentaenoic acid; DHA, docosahexaenoic acid; LA, linoleic acid; TNF, Tumor Necrosis Factor; IL-6, Interleukin 6; PF4, Platelet Factor 4; NAP-2, Neutrophil-Activating Peptide-2.

**Figure:**
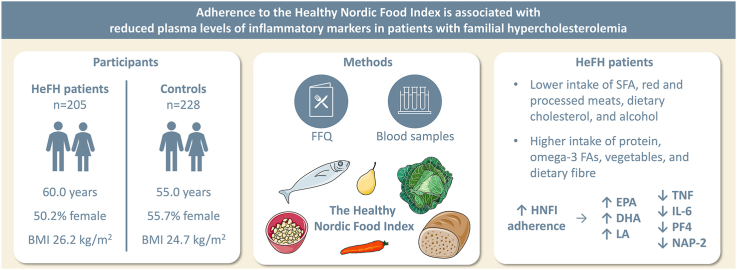

## Teaser

We assessed the dietary pattern in relation to the Healthy Nordic Food Index (HNFI) in adults with and without heterozygous familial hypercholesterolemia (HeFH) and examined the associations between HNFI score and biomarkers related to cardiovascular disease in the HeFH group.•The HeFH patients had a higher intake of nutrients and food groups often considered healthy, and a lower intake of nutrients and food groups often considered unhealthy, than the controls•In the HeFH group, increasing adherence to the HNFI was associated with increasing plasma levels of omega-3 and omega-6 fatty acids, and reduced levels of inflammatory markers.

## 1 Introduction

Cardiovascular diseases (CVDs) are the leading cause of death on a global level [1]. Most CVD deaths are due to myocardial infarctions and ischemic strokes [1], with atherosclerosis as the predominant cause [2]. Low-density lipoprotein (LDL) is the principal carrier of cholesterol (C) in human plasma and the primary driver of atherogenesis [2]. Lowering LDL-C levels reduces the risk of coronary heart disease (CHD) events [3], and replacing saturated fatty acids (SFAs) with the appropriate macronutrients decreases CHD risk [4]. When isocalorically substituted for SFAs, using polyunsaturated fatty acids (PUFAs), monounsaturated fatty acids (MUFAs), and whole grain carbohydrates is associated with a 25 %, 15 %, and 9 % decrease in CHD risk, respectively [5].

In nutrition research, the traditional approach has been to assess associations between single foods or nutrients, and clinical risk factors or health outcomes. Since the early 2000s, dietary pattern analysis has emerged as an alternative approach [6]. Dietary patterns and thus dietary quality may be assessed using dietary indices like the Healthy Nordic Food Index (HNFI) [7]. The HNFI is based on traditional Nordic foods demonstrated to have beneficial effects (i.e., fish, root vegetables, cabbages, apples and pears, whole grain rye bread, and oatmeal). Previous publications have found increasing HNFI score to be inversely associated with risk of both mortality [7,8], myocardial infarction [9], and stroke [10].

Inflammation is considered one of the primary causes of atherosclerotic aggravation, interacting in a bidirectional manner with lipid pathology [11]. C-reactive protein (CRP) predicts atherosclerotic CVD risk [12], and continuous expression of pro-inflammatory cytokines contributing to endothelial cell activation and recruitment of phagocytes in the atherosclerotic plaque, sustains inflammation and drives the atherosclerotic process further [2]. Platelets are mostly known as mediators of thrombus formation, but evidence suggests that they are also potent inflammatory cells interacting with among others monocytes and endothelial cells, further promoting atherogenesis [13,14].

Familial hypercholesterolemia (FH) is an inherited disease associated with hypercholesterolemia from the first year of life and increased risk of premature CVD [15]. In most cases, FH is caused by a mutation in the genes encoding the LDL receptor, apolipoprotein B (ApoB), or proprotein convertase subtilisin/kexin type 9 (PCSK9), leading to decreased hepatic uptake of LDL and consequently high levels of LDL-C in the circulation [15,16]. Dietary treatment is recommended as part of FH treatment in addition to lipid lowering treatment [15], but few studies have examined the dietary habits among FH patients and more data are needed on the diet in these patients [17] and their adherence to the dietary advice given as part of treatment. Moreover, data on the effect of the diet beyond that on lipids (i.e., effect on plasma fatty acids [FAs] and effect on inflammatory markers) are scarce or even lacking in patients with FH.

The aim of this study was to assess the dietary pattern in relation to the HNFI in well-treated (both in primary and secondary prevention) adults with HeFH and control subjects. Furthermore, we aimed to examine the associations between dietary quality and blood biomarkers related to cardiovascular disease, including inflammatory markers, in adults with HeFH.

## 2 Methods

### 2.1 Study design and participants

The FH subjects were recruited through the outpatient Lipid Clinic, Oslo University Hospital, and were participants in two previously conducted cross-sectional studies at the Department of Nutrition, University of Oslo, Norway [18,19]. The studies included adults with a definite FH diagnosis (either genetic and heterozygous [HeFH], or clinical) aged ≥18 years and ≥65 years, respectively. Clinical diagnosis was defined by a score >8 according to the Dutch Lipid Clinic Network diagnostic criteria for FH [16]. Exclusion criteria were diabetes mellitus type 1, severe illness (e.g., infection, autoimmune disease, or cancer) or ongoing treatment of such illness, uncontrolled hypertension, pregnancy, or lactation. The HeFH subjects included patients in both primary and secondary prevention, and all continued their use of lipid lowering treatment during the period prior to data collection.

The control subjects were healthy participants aged 30–70 years attending the screening visit of a study previously conducted at the Department of Nutrition, University of Oslo, Norway [20]. To be invited to the screening visit, the participants had to be healthy (i.e., free from chronic disease), weight stable (i.e. maximum weight change of ±5 % in the past three months), and with a body mass index (BMI) in the range 20–31 kg/m^2^. Other exclusion criteria were self-reported elevated blood pressure, glucose, cholesterol, or triglyceride levels, and use of cholesterol-lowering medication. Subjects who reported using omega-3 PUFA supplements on a regular basis were asked to refrain from taking the supplements for at least two weeks before the screening visit.

Compared to the Norwegian general population, our control subjects had slightly elevated total cholesterol levels. However, when looking at their dietary habits, they had similar intake of saturated fat compared to the general Norwegian population shown in the latest national dietary survey [21]. Thus, in relation to dietary habits, we believe that they represent a valid reference population for the HeFH group.

The study protocols were approved by the Regional Ethics Committee for Medical Research in South East Norway. All participants gave a written informed consent. The study was conducted in accordance with the principles of the Declaration of Helsinki.

### 2.2 Assessment of dietary intake

Dietary intake was assessed using two self-administered semi-quantitative food frequency questionnaires (FFQs). In the FH studies, an FFQ with 256 questions was used. The control subjects filled out an adapted version of the same FFQ. The adapted version had 270 questions and included all the questions from the original version as well as extra questions about intake of herbs, spices, nuts, and more alternatives for jam and juice. Both FFQs were developed at the Department of Nutrition, University of Oslo, Norway. They were designed to capture the habitual dietary intake during the past 12 months, including both frequency and portion sizes. The FFQs have been validated and the details are described elsewhere [22,23].

### 2.3 Biochemical analyses

The HeFH patients gave a non-fasting blood sample, while the controls gave a fasting blood sample (≥12 h).

In the FH studies, standard biochemical analyses (i.e., total cholesterol, LDL-C, high-density lipoprotein [HDL]-C, apolipoprotein A [ApoA], ApoB, triglycerides [TAG], and glucose), α_1_-antitrypsin, fibrinogen, and CRP were analysed by standard methods at the Department of Medical Biochemistry, Rikshospitalet, Oslo University Hospital, Norway. Lipoprotein(a) (Lp(a)) was analysed using an immunoturbidimetric method from Roche Diagnostics at the same medical laboratory [19,24].

For the controls, total cholesterol, LDL-C, HDL-C, ApoA, ApoB, TAG, glucose, and CRP were measured by standard methods at a routine laboratory (Fürst Medical Laboratory, Oslo, Norway) [20].

The inflammatory/anti-inflammatory markers interleukin (IL)-6, IL-8, IL-10, tumor necrosis factor (TNF), interferon (IFN)-γ, and transforming growth factor (TGF)-β were analysed using commercial kits from Meso Scale Discovery by the commercial laboratory Vitas Analytical Services. The platelet derived chemokines/cytokines Platelet Factor 4 (PF4/CXCL4), Neutrophil Activating Protein 2 (NAP-2/CXCL7), Regulated upon Activation Normal T-cells Expressed and Secreted (RANTES/CCL5), and soluble CD40 ligand (sCD40L) were analysed by enzyme-linked immunosorbent assay from RnD Systems, Minneapolis, USA [18]. Inflammatory and platelet markers were only measured for the HeFH patients.

Total plasma FA composition was analysed by GC-flame ionization detector analysis at the commercial laboratory Vitas Analytical Services [24] as previously described [20]. The concentration of the individual FAs is presented as percentage of total FAs (percent of total FA methyl esters; %FAME).

### 2.4 Operationalization of the Healthy Nordic Food Index

The HNFI scores were computed as described in the original publication by Olsen et al. [7] Some adaptions were made due to cultural differences between Denmark and Norway, and the availability of data from the FFQs used in this study. In Norway, bread based on whole grain wheat, or a combination of whole grains, is more common to eat than rye bread. Thus, the original “Whole grain rye bread” category was replaced by “Whole grain bread”. The original “Oatmeal” category was replaced by “Breakfast cereals/oatmeal” because oats and other whole grains are commonly consumed as part of breakfast cereals like muesli, in Norway. The operationalization of the HNFI in this study is shown in Supplementary Table 1.

Sex-specific medians for the healthy control group were used as cut-off values for scoring. For each HNFI component, a score of zero was given to participants with an intake below the sex-specific median intake, while participants with an intake above or equal to the sex-specific median were given one point. The points for each HNFI component were then summed up to provide a total HNFI score for each participant.

### 2.5 Statistical methods

The statistical analyses were performed using SPSS Statistics version 28 and 29 (IBM, New York, USA).

Participants reporting energy intakes below 4000 kJ or above 20 000 kJ [25] were considered under- or over-reporters, respectively, and were excluded from the statistical analyses.

Median values with 25th and 75th percentiles were used to present central tendencies for continuous variables which were not normally distributed. Categorical data were presented as number (n) and percent (%). For continuous variables which were not normally distributed, Mann-Whitney U tests were used to analyse for differences between groups. For categorical variables, Chi-squared tests were used. Fisher's exact test was used when the preconditions for the Chi-squared test were not fulfilled. Simple and multiple linear regression was used to assess differences in dietary intake between groups. Both unadjusted and adjusted analyses were performed. In the adjusted analyses, the association between dietary intake and study group (i.e. HeFH or control) was corrected for age, BMI, and sex. Sensitivity analyses were performed using a censored regression model (Tobit model). Censored regression was used because the outcome variables were dietary variables which can take the value zero.

Two-sided *P*-values <0.05 were considered statistically significant.

Some variables (i.e., Lp(a), CRP, fibrinogen, IL-6, and IL-10) were corrected due to limitations in the resolution of the laboratory instruments used in the biochemical analyses. For the regression analyses, values equal to the limit of detection (LOD) for the laboratory instruments were replaced by imputed values below the LOD, for Lp(a) and CRP. The details of the corrections and imputations are described in ***Supplementary Methods.***

Simple and multiple linear regression was used to assess associations between dietary score and biochemical measurements. For right-skewed data, logarithmic transformations (the natural logarithm, ln) were applied to the variables prior to regression analysis. Due to the interpretation of regression coefficients when the outcome variable is log-transformed, the effect estimate is given in percent for the log-transformed outcome variables. The effect estimate corresponds to the change in percent for the outcome variable, for every 1-point increase in HNFI score, when all other predictors in the regression model are held constant.

In the adjusted models, the associations between dietary score and biochemical measurements were corrected for sex, age, BMI, and smoking status.

To be able to present the results of the linear regression analyses in forest plots, the biochemical variables were standardized before regression analysis to be comparable on the same scale. This was done by subtracting the mean value and dividing by the standard deviation, for each variable. In the forest plots, the effect estimates are given in number of standard deviations for all variables.

## 3 Results

### 3.1 Participant characteristics

A total of 205 patients with HeFH and 228 control subjects were included in the study (Supplementary Fig. 1).

The HeFH group and the control group were comparable concerning sex distribution (50.2 % and 55.7 % women, respectively), but the HeFH group was older (median age 60.0 years vs. 55.0 years, respectively; *p* = 0.001) and had a higher BMI (median BMI 26.2 kg/m^2^ vs. 24.7 kg/m^,^ respectively; *p* = 0.03) than the control group (Table 1). There was no significant difference between the groups regarding proportion of current smokers. Among the HeFH patients, 25.9 % (n = 53) had experienced a CVD event. The HeFH patients had significantly lower levels of both total cholesterol, LDL-C, HDL-C, ApoA, and ApoB compared to the control group, most probably reflecting that the majority of the HeFH patients used lipid lowering treatment (95.6 %), with statins being the most prevalent choice.

**Table 1:** Descriptive characteristics of the study population.

	n	HeFH group	n	Control group	*p*-value^a^
**Age (years)**	205	60.0 (41.0, 70.0)	228	55.0 (46.0, 61.0)	**0.001**^b^
**Female, n (%)**	205	103 (50.2)	228	127 (55.7)	0.29^c^
**BMI (kg/m**^**2**^**)**	205	26.2 (22.6, 28.7)	226	24.7 (22.6, 27.4)	**0.03**^b^
**Current smoking, n (%)**	205	18 (8.8)	214	31 (14.5)	0.09^c^
**FH diagnosis, n (%)**	205				
*Genetic*		200 (97.6)			
*Clinical*		5 (2.4)			
**Lipids**					
Total cholesterol (mmol/L)	205	4.4 (3.9, 5.3)	227	6.4 (5.8, 7.0)	**<0.001**^b^
LDL-C (mmol/L)	205	2.6 (2.0, 3.2)	227	4.0 (3.4, 4.5)	**<0.001**^b^
HDL-C (mmol/L)	205	1.5 (1.2, 1.8)	226	1.6 (1.3, 1.9)	**0.002**^b^
ApoA (g/L)	205	1.5 (1.3, 1.7)	226	1.6 (1.4, 1.7)	**0.01**^b^
ApoB (g/L)	205	0.9 (0.8, 1.1)	226	1.3 (1.1, 1.4)	**<0.001**^b^
TAG (mmol/L)	205	1.0 (0.8, 1.5)	226	1.1 (0.8, 1.6)	0.22^b^
**Metabolic markers**					
Glucose (mmol/L)	201	5.3 (5.0, 5.9)	227	5.3 (5.0, 5.6)	0.13^b^
**Inflammatory markers**					
CRP (mg/L)	122	0.6 (0.6, 0.9)	227	1.1 (0.6, 1.9)	**<0.001**^b^
**LLT use, n (%)**	205	196 (95.6)			
*Statins*	205	191 (93.2)			
*PCSK9 inhibitor*	205	42 (20.5)			
*Colesevelam*	205	37 (18.0)			
*Ezetimibe*	205	149 (72.7)			
**Acetylsalisylic acid, n (%)**	205	92 (44.9)			
**Hypertension medication, n (%)**	108	14 (13.0)	228	25 (11.0)	0.72^c^

Data are presented as medians and 25th and 75th percentiles for continuous variables, and number (n) and percent (%) for categorical variables.

HeFH, heterozygous familial hypercholesterolemia; BMI, body mass index; FH, familial hypercholesterolemia; LDL-C, low-density lipoprotein cholesterol; HDL-C, high-density lipoprotein cholesterol; ApoA, apolipoprotein A; ApoB, apolipoprotein B; TAG, triacylglycerol; CRP, C-reactive protein; LLT, lipid-lowering therapy; PCSK9, proprotein convertase subtilisin/kexin type 9.

a p-values <0.05 are considered statistically significant (marked in bold).

b Mann-Whitney *U* test.

c Chi-Squared test.

Plasma FA levels in the HeFH and control group are presented in Supplementary Table 2, showing lower docosahexaenoic acid (DHA, C22:6-n3) and linoleic acid (LA, C18:2n-6) and higher arachidonic acid (AA, C20:4n-6) in the HeFH group.

### 3.2 Dietary intake

When adjusting for age, BMI, and sex, the HeFH patients had a lower intake of total fat and saturated fat than the control group (32.6 energy percent (E%) vs. 34.9 E%, and 9.6 E% vs. 12.0 E%, respectively; *p* < 0.001 for both) (Table 2). They also had a higher intake of MUFA and n-3 PUFAs than the controls (*p* < 0.001 for both), but a lower intake of total PUFAs and n-6 PUFAs fatty acids (*p* = 0.01 for both). The intake of added sugar was similar in both groups, but the HeFH subjects ate more carbohydrates and dietary fibre than the control group (43.2 E% vs. 41.0 E%, and 2.8 E% vs. 2.4 E%, respectively; *p* < 0.001 for both). Furthermore, the HeFH patients reported a higher protein intake than the control subjects (17.9 E% vs. 16.7 E% respectively; *p* < 0.001), but a lower intake of alcohol and dietary cholesterol (*p* < 0.001 for both).

**Table 2:** Energy and nutrient intakes of the study population.

	HeFH group (n = 205)	Control group (n = 228)	Unadjusted *p-*value^a^^,^^b^	Adjusted *p-*value^a^^,^^b^^,^^c^^,^^d^
**Energy (kJ)**	9503.8 (9152.8, 9854.8)	11 472.2 (11 066.7, 11 877.7)	**<0.001**	**<0.001**
**Protein (E%)**	17.9 (17.6, 18.2)	16.7 (16.4, 17.0)	**<0.001**	**<0.001**
**Fat (E%)**	32.6 (31.9, 33.2)	34.9 (34.1, 35.8)	**<0.001**	**<0.001**
**SFA (E%)**	9.6 (9.3, 9.9)	12.0 (11.7, 12.4)	**<0.001**	**<0.001**
**TFA (E%)**	0.2 (0.2, 0.2)	0.4 (0.4, 0.4)	**<0.001**	**<0.001**
***cis*-MUFA (E%)**	12.8 (12.4, 13.1)	11.9 (11.6, 12.2)	**<0.001**	**<0.001**
***cis*-PUFA (E%)**	7.1 (6.9, 7.4)	7.6 (7.3, 7.9)	**0.01**	**0.01**
***n-3* PUFA (E%)**^e^	1.9 (1.5, 2.4)	1.5 (1.2, 2.1)	**<0.001**	**<0.001**
***n-6* PUFA (E%)**^e^	4.8 (4.1, 5.5)	5.1 (4.2, 6.0)	**0.01**	**0.01**
**Carbohydrates (E%)**	43.2 (42.4, 44.0)	41.0 (40.0, 41.9)	**<0.001**	**<0.001**
**Dietary fibre (E%)**	2.8 (2.7, 2.9)	2.4 (2.3, 2.5)	**<0.001**	**<0.001**
**Sugar (E%)**^e^	4.2 (2.7, 6.3)	4.5 (2.9, 6.4)	0.61	0.80
**Alcohol (E%)**^e^	2.5 (1.1, 5.0)	3.7 (1.5, 6.7)	**<0.001**	**<0.001**
**Cholesterol (mg)**	281.0 (264.5, 297.5)	342.6 (325.4, 360.0)	**<0.001**	**<0.001**

Data are presented as means and 95 % confidence intervals, unless otherwise is stated.

HeFH, heterozygous familial hypercholesterolemia; E%, percent of energy; SFA, saturated fatty acids; TFA, trans-unsaturated fatty acids; MUFA, mono-unsaturated fatty acids; PUFA, poly-unsaturated fatty acids.

a p-values <0.05 are considered statistically significant (marked in bold).

b Linear regression models.

c n = 226 in the control group due to n = 2 missing data on BMI.

d Adjusted for age, BMI, and sex.

e Data are presented as medians and 25th and 75th percentiles.

Concerning food groups, the HeFH patients had a significantly higher intake of vegetables and a lower intake of red and processed meat than the controls (333.5 g/d vs. 254.2 g/d, and 68.1 g/d vs. 83.6 g/d, respectively; *p* < 0.001 for both) (Supplementary Table 3). Furthermore, they had a higher intake of low-fat cheese (*p* < 0.001). In addition, they reported a lower intake of eggs than the controls, but a higher intake of unsalted nuts (p = 0.02 and p = 0.01, respectively). The intake of whole grain bread and cereals was lower in the HeFH group compared to the control group (120.0 g/d vs. 90.0 g/d in the HeFH group, *p* = 0.004), but the intake of fruits and berries, fish, low-fat milk, and full-fat cheese was similar in both groups.

Food group intakes for men and women in both groups are presented in Supplementary Table 4.

When comparing dietary intake in the HeFH group and the control group, sensitivity analyses using censored regression models (Tobit models) showed results similar to the linear regression models. Thus, the results (p-values) from the linear regression models are presented in Supplementary Table 3 and Supplementary Table 4.

### 3.3 Healthy Nordic Food Index scores

When adjusting for age, BMI, and sex, both the HeFH group and the control group had a moderate dietary quality as measured by the HNFI (median score 3 out of 6 points) (Table 3). When looking at each index component separately, the HeFH patients had a higher median intake of fish, root vegetables, and whole grain cereals than the controls, but a lower intake of whole grain bread (Table 3). There was no difference regarding intake of cabbage or apples and pears between the groups.

**Table 3:** Total Healthy Nordic Food Index score and daily intake of the index components in the study population.

	HeFH group			Control group			Unadjusted *p-*value^a^^,^^b^			Adjusted *p-*value^a^^,^^b^		
	Men (n = 102)	Women (n = 103)	All (n = 205)	Men (n = 101)	Women (n = 127)	All (n = 228)	Men	Women	All	Men^c^^,^^d^	Women^c^^,^^e^	All^f^
**Fish, g/d**	78.5 (54.0, 115.3)	70.2 (47.2, 104.6)	74.0 (49.7, 110.5)	63.6 (39.8, 98.1)	61.5 (38.0, 89.4)	62.4 (38.3, 95.0)	**0.02**	**0.04**	**0.001**	0.14	**0.03**	**0.01**
**Root vegetables, g/d**	40.1 (16.2, 82.2)	58.2 (31.3, 103.8)	56.8 (24.4, 85.8)	26.7 (13.3, 48.3)	40.6 (23.1, 67.8)	31.8 (17.8, 57.5)	**<0.001**	**0.001**	**<0.001**	**0.01**	**0.003**	**<0.001**
**Cabbage, g/d**	27.0 (11.6, 64.2)	41.0 (17.9, 87.5)	35.6 (15.3, 68.9)	30.8 (14.0, 51.5)	37.8 (20.7, 60.1)	33.1 (16.7, 58.7)	0.84	0.09	0.21	0.66	0.09	0.12
**Apples and pears, g/d**	45.7 (8.9, 88.4)	54.7 (17.8, 108.7)	45.7 (17.2, 101.4)	54.0 (17.0, 84.0)	70.5 (27.0, 112.5)	64.5 (21.0, 106.5)	0.45	0.30	0.15	0.30	0.40	0.18
**Whole grain bread, g/d**	109.5 (13.0, 180.0)	78.0 (26.0, 116.0)	90.0 (26.0, 148.0)	120.0 (28.0, 194.0)	127.0 (44.0, 187.0)	120.0 (30.0, 187.0)	0.30	**<0.001**	**0.001**	0.51	**<0.001**	**0.002**
**Whole grain cereals, g/d**	14.0 (0.0, 74.3)	24.5 (4.6, 60.3)	24.3 (0.0, 71.8)	7.8 (0.0, 34.5)	14.0 (0.0, 46.5)	10.9 (0.0, 41.2)	0.01	**0.02**	**<0.001**	**0.001**	**0.01**	**<0.001**
**Healthy Nordic Food Index score, points**	3.0 (2.0, 5.0)	3.0 (2.0, 4.0)	3.0 (2.0, 4.0)	3.0 (2.0, 4.0)	3.0 (2.0, 4.0)	3.0 (2.0, 4.0)	0.49	0.78	0.72	0.80	0.87	0.82

Data are presented as medians and 25th and 75th percentiles.

HeFH, heterozygous familial hypercholesterolemia; g/d, grams per day.

^g^ n = 226 in the control group due to n = 2 missing data on BMI.

a p-values <0.05 are considered statistically significant (marked in bold).

b Linear regression models.

c Adjusted for age and BMI.

d n = 100 in the control group due to n = 1 missing data on BMI.

e n = 126 in the control group due to n = 1 missing data on BMI.

f Adjusted for age, BMI, and sex.

When comparing men and women separately, the difference in intake of fish and whole grain bread was only significant among the women.

When comparing intake of HNFI components in the HeFH group and the control group, sensitivity analyses using censored regression models (Tobit models) showed results similar to the linear regression models. Thus, the results (p-values) from the linear regression models are presented in Table 3.

**Fig. 1:**
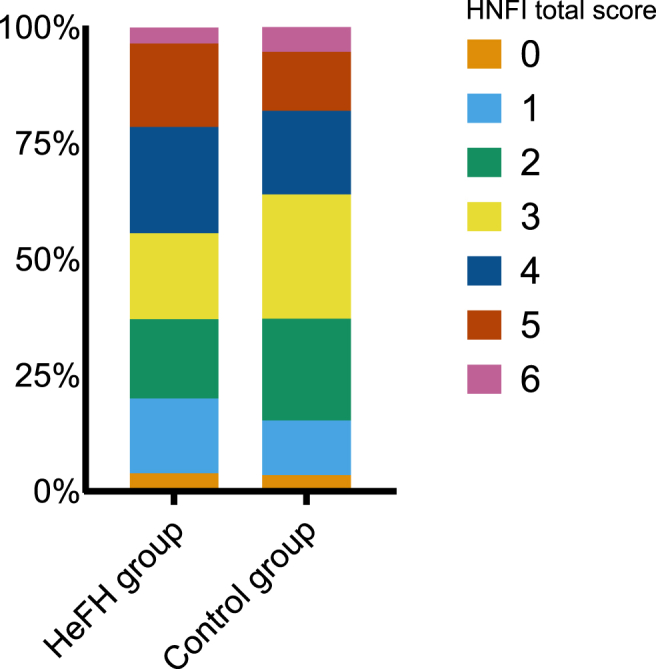
Distribution of Healthy Nordic Food Index total scores in the study population Abbreviations: HeFH, familial hypercholesterolemia; HNFI, Healthy Nordic Food Index.

Concerning the distribution of total HNFI scores, a higher proportion of the HeFH group had either low (0–1 points) or high (5–6 points) scores compared to the control group (Fig. 1). Consequently, the proportion of participants with medium (2–4 points) scores was higher in the control group. In both groups, the majority of the participants had medium scores (2–4 points).

### 3.4 Associations between Healthy Nordic Food Index score and blood biomarkers in patients with HeFH

**Fig. 2:**
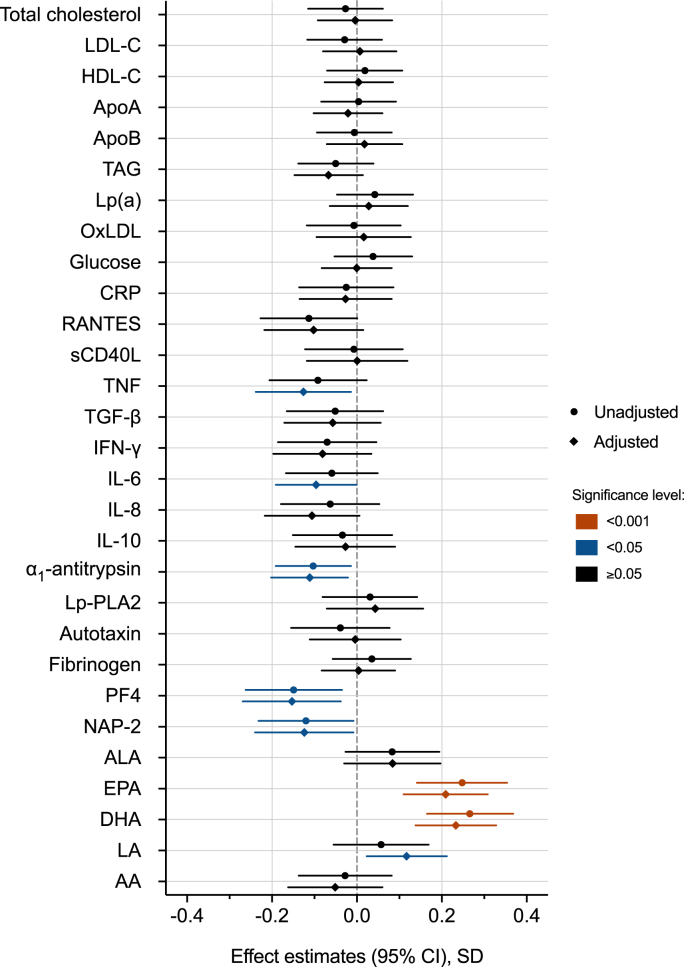
Associations between Healthy Nordic Food Index score and blood biomarkers in the HeFH group The red and blue symbols illustrate statistically significant associations (p < 0.05). The adjusted models are adjusted for sex, age, BMI, and smoking status. Abbreviations: LDL-C, low density lipoprotein cholesterol; HDL-C, high density lipoprotein cholesterol; ApoA, apoliprotein A; ApoB, apolipoprotein B; TAG, triacylglycerol; Lp(a), lipoprotein(a); OxLDL, oxidized LDL; CRP, C-reactive protein; RANTES, Regulated upon Activation Normal T-cells Expressed and Secreted; sCD40L, soluble CD40 ligand; TNF, Tumor Necrosis Factor; TGF-β, Transforming Growth Factor β_1_; IFN-γ, Interferon γ; IL-6, Interleukin 6; IL-8, Interleukin 8; IL-10, Interleukin 10; Lp-PLA2, Lipoprotein-associated phospholipase A2; PF4, Platelet factor 4; NAP-2, Neutrophil-Activating Peptide-2; ALA, α-Linolenic Acid; EPA, Eicosapentaenoic acid; DHA, Docosahexaenoic acid; LA, Linoleic acid, AA, Arachidonic acid. (For interpretation of the references to colour in this figure legend, the reader is referred to the Web version of this article.)

Associations between HNFI score and blood biomarkers were only assessed in the HeFH group. The blood levels of lipids, inflammatory markers, and platelet markers in the HeFH group are presented in Supplementary Table 5. When adjusted for sex, age, BMI, and smoking status, increasing HNFI score was significantly associated with an increase in plasma levels of the n-3 PUFAs eicosapentaenoic acid (EPA) and DHA, and the n-6 PUFA LA. Conversely, increasing HNFI score was significantly associated with a decrease in the levels of the inflammatory cytokines TNF and IL-6, the platelet derived chemokines PF4 and NAP-2, as well as α_1_-antitrypsin levels (Fig. 2, Supplementary Table 6).

## 4 Discussion

To the best of our knowledge, this is the first study to assess dietary pattern and associations between dietary pattern and FAs and CVD-related inflammatory biomarkers in an HeFH population.

Despite dietary counselling being an important part of the treatment of patients with FH, few studies have examined the dietary habits among FH patients [24,[26], [27], [28], [29]]. In children with FH, the intake of saturated fat is positively associated with total and LDL-C [29], but as many as 30 % of children and young adults with FH have adherence issues relating to following the recommended treatment [30]. In this project we aimed, in a cohort of well-treated HeFH patients followed-up at a specialized lipid clinic, to specifically study the dietary pattern, and not only the macronutrient composition. More importantly, we aimed to study whether adherence to a healthy dietary pattern was associated with other CVD risk markers beyond the known and expected effects on lipid profile (i.e., FAs and in particular inflammatory markers). This has to our knowledge not been described before, and our data suggest that a healthy diet has a role beyond the effect on lipid lowering. This emphasizes the importance of dietary counselling in the treatment of FH patients.

We found that the HeFH patients in general had a healthier diet than the controls, with lower intake of saturated fat and red and processed meat, and higher intake of vegetables. Both groups had a moderate HNFI score, with no difference between the groups. In HeFH patients, we found that increasing HNFI score was positively associated with plasma EPA, DHA, and LA levels, and negatively associated with blood levels of several inflammatory and platelet-derived markers. This suggests that adherence to an overall healthy dietary pattern might be beneficial for HeFH patients independent of the cholesterol-lowering effect of the diet.

We found no association between HNFI score and plasma levels of total and LDL-C. This was not surprising as 96 % of the patients with HeFH were on current statin treatment. However, the observed increase in HNFI score significantly associated with an increase in plasma levels of the FAs EPA, DHA and LA, and with a decrease in the levels of inflammatory cytokines, indicates that the diet confer an additional benefit of statin-therapy on the FA profile and inflammation. Our data are supported by a recent study from the Copenhagen general population study [31] where they showed that non-adherence to dietary guidelines was associated with an atherogenic lipid and inflammatory profile. They showed that replacing a dietary score with the well-known risk factor non-HDL cholesterol in the risk charts, resulted in similar risk estimates, underscoring the importance and potential of dietary treatment in the prevention of CVD. However, our study is first to show a similar pattern in well-treated HeFH patients.

When comparing the dietary intake between the groups, we observed that the HeFH group had a lower consumption of macronutrients and food groups that are typically deemed unhealthy, such as saturated fat, alcohol, dietary cholesterol, and red and processed meats. In contrast, their intake of macronutrients and food groups commonly associated with a healthy diet, such as omega-3 fatty acids, protein, vegetables, and dietary fibre, was higher. This suggests that Norwegian adults with HeFH who receive treatment at specialized lipid clinics, eat healthier than controls. This is in accordance with a previous study conducted among Norwegian children and young adults with FH, which showed that the FH subjects had healthier food choices compared with non-FH children with respect to dietary fat sources [28]. Also, results from the SAFEHEART study show that Spanish adults with FH report healthier dietary and lifestyle habits than their non-affected family members [27].

A previous study showed that 87 % of all FH adults treated at Norwegian lipid clinics have received dietary counselling [32], and that the plasma lipid profile was improved after dietary counselling in children and adolescents with FH [29]. Moreover, dietary advice has been showed to improve blood lipids and reduce CHD incidence and CHD mortality in both primary [33] and secondary prevention [34] in Norwegian men. However, a Cochrane review from 2014 concluded that data was inadequate on the effectiveness of dietary treatment on CHD events and mortality in FH subjects [17].

Despite the explorative nature of the analyses; the results of this study raise the hypothesis that a healthy dietary pattern can reduce blood levels of inflammatory and platelet-derived inflammatory markers in HeFH patients. FH subjects are characterized by increased inflammation compared to controls [35], as well as increased platelet activation and an underlying pro-coagulant state [2]. A previous study [18] showed that the FH subjects had higher plasma levels of platelet-derived inflammatory cytokines compared to controls. Age of onset of clinically manifested CHD varies greatly even among FH patients with similar phenotype or genotype, and can possibly be explained by other risk factors [36]. Inflammation might be one of the factors influencing the progression and development of atherosclerosis in FH patients [35]. The role of platelet-derived cytokines in FH is yet unclear, but platelet-derived cytokines have been found present in human atherosclerotic plaque [37] and mouse models have shown PF4 to aggravate atherosclerotic lesions [38].

In this study, increasing HNFI score was also associated with higher plasma levels of EPA, DHA, and LA. EPA and DHA are long-chained marine n-3 PUFAs, giving rise to resolvins and protetctins which are pro-resolving mediators found to have anti-inflammatory effects. These lipid substances may reduce inflammation and attenuate atherogenesis [39]. Consumption of DHA is associated with reduced endothelial expression and plasma levels of IL-6 and TNF [40,41], both of which were found to be inversely associated with increasing HNFI score in this study. Higher total plasma n-3 PUFA levels are also found to be associated with lower levels of plasma pro-inflammatory markers like IL-6 and TNF [41], and in the present study, increasing HNFI score was associated with higher levels of DHA and EPA, and lower levels of these cytokines. Furthermore, Pischon et al. [42] found that intake of omega-3 and omega-6 PUFAs in combination was associated with lower levels of inflammatory cytokines, and that the association was stronger than for omega-3 PUFAs alone.

A systematic review from 2013 [43] found that among non-FH subjects, adherence to a healthy dietary pattern (i.e., a fruit and vegetable based diet) was associated with lower levels of inflammatory markers. In contrast, “Western” dietary patterns (i.e., dietary patterns dominated by red and processed meats, fried potatoes, refined grains, high sugar intake, and high fat dairy) were associated with increased levels of inflammatory markers. This supports our findings, which suggest that higher adherence to a healthy dietary pattern with high intakes of fruits and vegetables is associated with lower levels of several inflammatory markers and platelet-derived cytokines in HeFH individuals.

Our results from these explorative analyses add to the hypothesis that a healthy Nordic diet has anti-inflammatory potential. As described in the original publication of the HNFI [7], the foods included in the HNFI belong to food groups with established health-beneficial effects including reduced incidence of CVD. However, it is worth noting that high HNFI score was related to a general healthy lifestyle in the original publication where other lifestyle factors were assessed, indicating that residual confounding may influence the associations between HNFI and health outcomes [7].

Due to the effectiveness of statins on lowering cholesterol, dietary treatment of FH has received less emphasis lately [44]. Furthermore, when focusing on the cholesterol-lowering potential of diet in relation to clinical outcomes in FH patients, evidence is inconclusive [17], and data on diet in relation to clinical events in FH is hard to obtain due to both ethical and practical considerations. A recent study found that like the general population, FH patients are likely to benefit from adherence to a healthy lifestyle (i.e., healthy diet, physical activity, no smoking, and normal bodyweight) to reduce CHD risk, despite a minimal association between adherence to a healthy lifestyle and LDL-C levels [45]. Among the FH subjects, having a healthy lifestyle was associated with an 86 % lower risk of CHD compared to having an unhealthy lifestyle [45]. Similarly, a review assessing associations of dietary patterns with atherosclerosis risk biomarkers (i.e., LDL-C, ApoB, and CRP) in FH patients, found beneficial effects of a healthy dietary pattern combined with an overall healthy lifestyle [46]. Although most of the included studies were limited by a cross-sectional design, the authors conclude that an overall healthy dietary pattern in line with the Mediterranean diet might even be more favourable to FH patients than primarily restricting SFA intake.

The main limitations of this study are the cross-sectional design and the measurement errors and types of bias associated with the dietary assessment method used. Although being validated, the FFQs used to assess dietary intake in this study were not specifically designed to measure intake of the index components of the HNFI. The adaptions made to the operationalization of the HNFI limits the comparability of the dietary quality to the dietary quality of other populations.

The cross-sectional study design limits this study to be hypothesis generating. The lack of Bonferroni correction increases the risk of type I errors in the multiple testing and must be considered as a limitation of the study. However, considering the explorative nature of this study, we have used the nominal p-values when assessing associations between HNFI score and blood biomarkers.

The lack of formal sample size calculation is also a limitation of this study. However, due to the explorative nature of the study and the limited number of studies available on dietary pattern among patients with FH, we consider the number of subjects in this study appropriate.

Finally, the use of a non-fasting blood sample in the HeFH subjects might have influenced plasma levels of biomarkers affected by recent dietary intake (e.g. TAG, plasma FAs, and glucose). However, the median values of these biomarkers in the HeFH group seem to be within the normal range of fasting values.

A major strength of this study is that several inflammatory markers previously found to be related to atherosclerosis were found to be associated with dietary quality. Furthermore, the study sample is likely to be representative of the general Norwegian adult HeFH population in treatment, as the participants were recruited through a lipid clinic and in general had received treatment including dietary counselling for years.

In conclusion, the present study suggests that Norwegian patients with HeFH receiving treatment at a specialized Lipid Clinic eat healthier than controls. In addition, in explorative analyses; adherence to a healthy dietary pattern is associated with higher plasma levels of n-3 and n-6 PUFAs, and lower levels of inflammatory markers, including markers of platelet-driven inflammation. This may suggest that adherence to an overall healthy dietary pattern might be beneficial for HeFH patients independent of the cholesterol-lowering effect of the diet.

## Authors’ contributions

Conceptualization: EBL, KR, KBH; Data curation: EBL, IN, TU, BH, PA, MPB; Formal analysis: EBL; Funding acquisition: KBH; Investigation: EBL, KR, KBH; Methodology: EBL, IN, TU, KR, KBH; Project administration: EBL, KR, KBH; Supervision: KR, KBH; Visualization: EBL; Writing – original draft: EBL, KR, KBH; Writing – review & editing: all authors.

## Financial support

The study was supported by the 10.13039/501100022293Throne-Holst Foundation for Nutrition Research, 10.13039/501100005366University of Oslo, Oslo, Norway, and the 10.13039/501100005366University of Oslo, Oslo, Norway.

## Declaration of competing interest

The authors declare the following financial interests/personal relationships which may be considered as potential competing interests: Dr. Bogsrud has received research grants and/or personal fees from 10.13039/100002429Amgen, 10.13039/100004339Sanofi, 10.13039/100004336Novartis, and 10.13039/100013220Ultragenyx, none of which are related to the present work. Dr. Retterstøl has received research grants and/or personal fees from 10.13039/100002429Amgen, 10.13039/100004336Novartis, and 10.13039/100004339Sanofi, none of which are related to the content of this manuscript. Dr. Holven has received research grants and/or personal fees from 10.13039/100004339Sanofi, none of which are related to the present work. The remaining authors have nothing to disclose.
