# Supplementary material for: Adherence to the Healthy Nordic Food Index is associated with reduced plasma levels of inflammatory markers in patients with heterozygous familial hypercholesterolemia
Source: Atheroscler Plus. 2024 Oct 24;58:38–45. doi: 10.1016/j.athplu.2024.10.003 (PMC11550195; doi:10.1016/j.athplu.2024.10.003)
Supplement: Multimedia component 1 [file mmc1.pptx]

## Slide 1
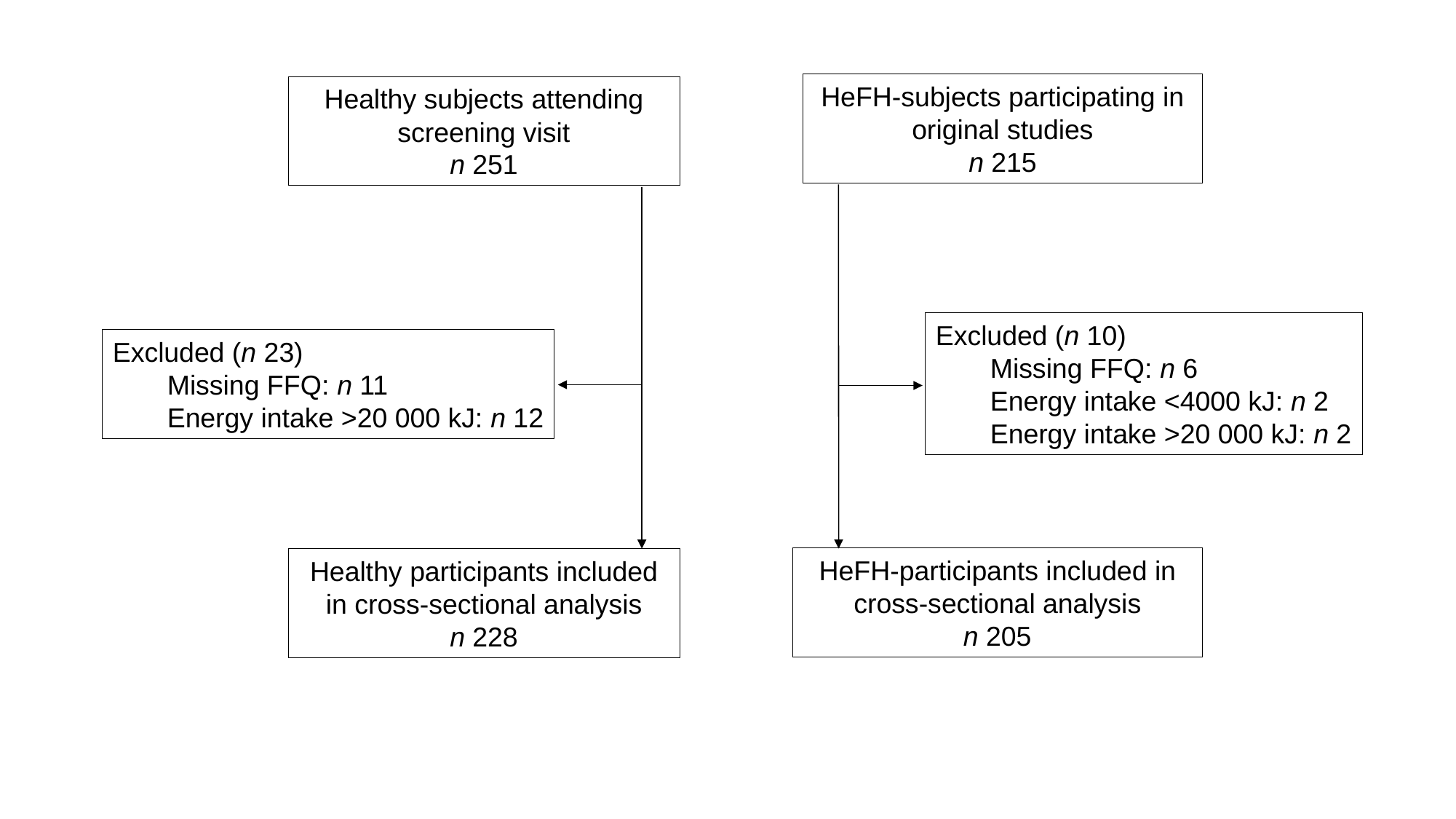

HeFH-subjects participating in
original studies
n 215
Healthy subjects attending screening visit
n 251
Excluded (n 10)
Missing FFQ: n 6
Energy intake <4000 kJ: n 2
Energy intake >20 000 kJ: n 2
Excluded (n 23)
Missing FFQ: n 11
Energy intake >20 000 kJ: n 12
HeFH-participants included in cross-sectional analysis
n 205
Healthy participants included in cross-sectional analysis
n 228
