# Supplementary material for: Adherence to the Healthy Nordic Food Index is associated with reduced plasma levels of inflammatory markers in patients with heterozygous familial hypercholesterolemia
Source: Atheroscler Plus. 2024 Oct 24;58:38–45. doi: 10.1016/j.athplu.2024.10.003 (PMC11550195; doi:10.1016/j.athplu.2024.10.003)
Supplement: Multimedia component 2 [file mmc2.docx]

**Supplementary Methods:**

Some variables were corrected due to limitations in the resolution of the laboratory instruments used in the biochemical analyses. For these variables, values below the limit of detection (LOD) of the instrument, were set equal to the LOD during lab analysis. This applies to Lp(a) (values <7.0 nmol/L were set to 7.0 nmol/L), CRP (values <0.6 mg/L were set to 0.6 mg/L), fibrinogen (values <0.1 g/L were set to 0.1 g/L), IL-6 (values <0.181 pg/mL were set to 0.181 pg/mL), and IL-10 (values <0.0894 pg/mL were set to 0.0894 pg/mL).

For the regression analyses, values equal to the LOD were replaced by imputed values below the LOD, for two of the corrected variables. This was the case for Lp(a) and CRP. Values below the LOD were only imputed when it helped improve the validity of the linear regression models (e.g., when the distribution of the standardized residuals was improved), and the imputations did not affect the associations. The imputations were made using a universal random number function in SPSS, which generates a random number between a given minimum and maximum value. The minimum and maximum values were set based on the accuracy of the values from the lab. If the values from the lab had a 0.01 accuracy, the minimum and maximum value were set to 0.01 and LOD-0.01, respectively. This was done to assure that the random number function in SPSS did not return zero nor the LOD. The imputed values were rounded to the same number of decimals as the values from the lab.

**Supplementary Table 1: Index components and scoring standards for the Healthy Nordic Food Index**

| **Healthy Nordic Food Index components** | **Cut-off values for scoring (sex-specific medians), g/day** | |
| --- | --- | --- |
|  | **Men** | **Women** |
| **Fish** | 63.6 | 61.5 |
| **Root vegetables** | 26.7 | 40.6 |
| **Cabbage** | 30.8 | 37.8 |
| **Apples and pears** | 54.0 | 70.5 |
| **Whole grain bread** | 120.0 | 127.0 |
| **Whole grain breakfast cereals/oatmeal** | 7.8 | 14.0 |

g/d, grams per day.

**Supplementary Figure 1: Flow diagram of the study participants**

Abbreviations: HeFH, heterozygous familial hypercholesterolemia; FFQ, food frequency questionnaire.

**Supplementary Table 2: Plasma fatty acid levels in the study population**

| **Fatty acid, % of FAME** | **HeFH group (n=122)** | **Control group (n=227)** | ***p*-value^a^** |
| --- | --- | --- | --- |
| ***n-3* PUFAs** |  |  |  |
| α-Linolenic acid, ALA (C18:3*n-3*) | 0.62 (0.49, 0.80) | 0.63 (0.54, 0.78) | 0.55 |
| Eicosapentaenoic acid, EPA (C20:5*n-3*) | 1.50 (1.03, 2.50) | 1.48 (1.07, 2.10) | 0.76 |
| Docosahexaenoic acid, DHA (C22:6*n-3*) | 2.40 (1.97, 3.00) | 3.07 (2.43, 3.68) | **<0.001** |
| ***n-6* PUFAs** |  |  |  |
| Linoleic acid, LA (C18:2*n-6*) | 23.45 (20.10, 26.40) | 26.68 (24.62, 28.66) | **<0.001** |
| Arachidonic acid, AA (C20:4*n-6*) | 8.10 (6.78, 9.33) | 5.30 (4.64, 5.95) | **<0.001** |

Data are presented as medians and 25^th^ and 75^th^ percentiles.

FAME, fatty acid methyl ester; HeFH, heterozygous familial hypercholesterolemia; PUFAs, poly-unsaturated fatty acids.

^a^ Mann-Whitney U tests. *p*-values <0.05 were considered statistically significant (marked in bold).

**Supplementary Table 3: Food group intakes of the study population**

| **Food group** | **HeFH group (n=205)** | **Control group (n=228)** | **Unadjusted *p-*value^a,b^** | **Adjusted *p-*value^a,b,c,d^** |
| --- | --- | --- | --- | --- |
| **Vegetables, g/d** | 333.5 (219.5, 457.7) | 254.2 (181.9, 359.5) | **<0.001** | **<0.001** |
| **Fruits and berries, g/d** | 180.7 (88.3, 269.6) | 184.4 (110.8, 262.2) | 0.83 | 0.98 |
| **Fatty fish, g/d** | 26.8 (16.5, 44.6) | 22.8 (14.0, 39.4) | 0.05 | 0.09 |
| **Lean and semi-fatty fish, g/d** | 15.6 (5.8, 30.2) | 17.2 (4.0, 32.1) | 0.33 | 0.12 |
| **Red and processed meat, g/d** | 68.1 (43.6, 94.7) | 83.6 (53.7, 110.0) | **0.001** | **<0.001** |
| **Whole grain bread and cereals, g/d** | 90.0 (6.5, 151.3) | 120.0 (30.0, 185.4) | **0.003** | **0.004** |
| **Low-fat milk, g/d** | 201.1 (100.0, 401.1) | 250.0 (15.8, 500.0) | 0.46 | 0.38 |
| **Low-fat cheese, g/d** | 4.0 (0.0, 17.5) | 0.0 (0.0, 1.75) | **<0.001** | **<0.001** |
| **Full-fat cheese, g/d** | 9.7 (3.4, 18.7) | 13.9 (9.2, 21.2) | 0.05 | 0.09 |
| **Eggs, g/d** | 18.3 (8.0, 25.9) | 21.6 (12.1, 32.8) | **0.02** | **0.02** |
| **Unsalted nuts, g/d** | 7.3 (2.3, 17.6) | 2.4 (0.0, 8.8) | **0.01** | **0.01** |

Data are presented as medians and 25^th^ and 75^th^ percentiles.

HeFH, heterozygous familial hypercholesterolemia; g/d, grams per day.

^a^ *p*-values <0.05 are considered statistically significant (marked in bold). ^b^ Linear regression models. ^c^ n=226 in the control group due to n=2 missing data on BMI. ^d^ Adjusted for age, BMI, and sex.

**Supplementary Table 4: Food group intakes for men and women in the HeFH and control group**

| **Food group** | **HeFH group** | | **Control group** | | **Unadjusted *p-*value^a,b^** | | **Adjusted *p-*value^a,b,c^** | |
| --- | --- | --- | --- | --- | --- | --- | --- | --- |
|  | **Men**  **(n=102)** | **Women**  **(n=103)** | **Men**  **(n=101)** | **Women**  **(n=127)** | **Men** | **Women** | **Men^d^** | **Women^e^** |
| **Vegetables, g/d** | 279.3  (188.2, 393.3) | 375.9  (278.0, 525.3) | 222.4  (151.5, 329.2) | 270.0  (200.8, 390.0) | **0.03** | **<0.001** | **0.01** | **<0.001** |
| **Fruits and berries, g/d** | 141.8  (73.1, 224.1) | 208.8  (114.5, 296.8) | 157.4  (82.0, 223.9) | 198.1  (127.0, 302.4) | 0.65 | 0.74 | 0.46 | 0.54 |
| **Fatty fish, g/d** | 25.2  (16.4, 48.6) | 27.6  (16.5, 44.2) | 22.8  (14.0, 40.0) | 22.8  (14.0, 37.5) | 0.19 | 0.17 | 0.36 | 0.10 |
| **Lean and semi-fatty fish, g/d** | 18.3  (7.1, 32.7) | 12.5  (4.1, 26.4) | 17.2  (3.2, 34.0) | 17.2  (5.8, 31.9) | 0.97 | 0.13 | 0.35 | 0.19 |
| **Red and processed meat, g/d** | 72.8  (52.3, 98.6) | 63.1  (38.1, 86.7) | 102.2  (67.8, 138.7) | 66.6  (43.4, 91.5) | **<0.001** | 0.27 | **0.001** | 0.09 |
| **Whole grain bread and cereals, g/d** | 129.7  (2.1, 180) | 60.3  (9.5, 107.3) | 120.0  (9.3, 224.0) | 120.0  (30.0, 180.0) | 0.32 | **<0.001** | 0.63 | **<0.001** |
| **Low-fat milk, g/d** | 202.5  (100.8, 429.4) | 200.0  (4.6, 400.0) | 259.8  (31.8, 512.6) | 134.8  (9.8, 259.8) | 0.14 | 0.83 | 0.34 | 0.86 |
| **Low-fat cheese, g/d** | 0.0  (0.0, 12.3) | 11.0  (0.0, 19.0) | 0.0  (0.0, 0.0) | 0.0  (0.0, 5.0) | **<0.001** | **<0.001** | **<0.001** | **<0.001** |
| **Full-fat cheese, g/d** | 10.2  (4.0, 20.0) | 9.6  (3.2, 18.0) | 16.0  (9.6, 22.1) | 12.7  (8.8, 19.1) | 0.12 | 0.21 | 0.44 | 0.22 |
| **Eggs, g/d** | 14.6  (7.1, 25.3) | 19.0  (9.1, 26.1) | 22.0  (12.3, 36.2) | 20.8  (11.9, 32.1) | **0.02** | 0.24 | 0.09 | 0.17 |
| **Unsalted nuts, g/d** | 5.3  (0.0, 15.7) | 8.0  (3.8, 19.3) | 1.2  (0.0, 6.1) | 3.3  (1.2, 19.2) | **0.01** | 0.14 | **0.02** | **0.11** |

Data are presented as medians and 25^th^ and 75^th^ percentiles.

HeFH, heterozygous familial hypercholesterolemia; g/d, grams per day.

^a^ *p*-values <0.05 are considered statistically significant (marked in bold). ^b^ Linear regression models. ^c^ Adjusted for age and BMI. ^d^ n=100 in the control group due to n=1 missing data on BMI. ^e^ n=126 in the control group due to n=1 missing data on BMI.

**Supplementary Table 5: Blood levels of lipids, inflammatory markers and platelet markers in the HeFH group**

|  | **n** | **HeFH group** |
| --- | --- | --- |
| **Lipids** |  |  |
| Lp(a) (nmol/L) | 202 | 66.0 (7.0, 214.0) |
| OxLDL (mU/L) | 122 | 58.1 (44.1, 69.0) |
| **Inflammatory markers** |  |  |
| RANTES (ng/mL) | 122 | 1.6 (1.4, 1.7) |
| sCD40L (ng/mL) | 122 | 0.4 (0.3, 0.6) |
| TNF (pg/mL) | 122 | 2.0 (1.6, 2.5) |
| TGF-β (ng/mL) | 122 | 2.7 (1.9, 3.5) |
| IFN-γ (pg/mL) | 122 | 4.1 (2.9, 7.0) |
| IL-6 (pg/mL) | 122 | 0.3 (0.2, 0.6) |
| IL-8 (pg/mL) | 122 | 3.3 (2.7, 4.4) |
| IL-10 (pg/mL) | 122 | 0.3 (0.2, 0.4) |
| α_1_-antitrypsin (g/L) | 205 | 1.2 (1.1, 1.3) |
| Lp-PLA2 (ng/mL) | 122 | 105.5 (76.4, 135.1) |
| Autotaxin (ng/mL) | 122 | 210.7 (180.7, 250.2) |
| **Platelet markers** |  |  |
| Fibrinogen (g/L) | 201 | 3.2 (2.8, 3.7) |
| PF4 (pg/mL) | 122 | 974.5 (820.0, 1186.8) |
| NAP-2 (pg/mL) | 122 | 273.5 (250.8, 297.0) |

Data are presented as medians and 25^th^ and 75^th^ percentiles.

HeFH, heterozygous familial hypercholesterolemia; Lp(a), lipoprotein(a); OxLDL, oxidized LDL; RANTES, Regulated upon Activation Normal T-cells Expressed and Secreted; sCD40L, soluble CD40 ligand; TNF, Tumor Necrosis Factor; TGF-β, Transforming Growth Factor β_1_; IFN-γ, Interferon γ; IL-6, Interleukin 6; IL-8, Interleukin 8; IL-10, Interleukin 10; Lp-PLA2, Lipoprotein-associated phospholipase A2; PF4, Platelet factor 4; NAP-2, Neutrophil-Activating Peptide-2.

**Supplementary Table 6: Associations between Healthy Nordic Food Index score and blood biomarkers in the HeFH group**

|  | **n** | **Unadjusted effect** | **95% CI** | ***p*-value^a^** | **Adjusted effect^b^** | **95% CI** | ***p*-value^a^** |
| --- | --- | --- | --- | --- | --- | --- | --- |
| **Lipids** |  |  |  |  |  |  |  |
| Total cholesterol, % | 205 | -0.80 | -3.34, 1.82 | 0.55 | -0.10 | -2.66, 2.53 | 0.92 |
| LDL-C, % | 205 | -1.49 | -5.92, 3.15 | 0.51 | 0.30 | -4.11, 4.92 | 0.88 |
| HDL-C, mmol/L | 205 | 0.01 | -0.03, 0.05 | 0.67 | 0.002 | -0.03, 0.04 | 0.92 |
| ApoA, g/L | 205 | 0.001 | -0.03, 0.03 | 0.93 | -0.01 | -0.03, 0.02 | 0.61 |
| ApoB, % | 205 | -0.20 | -3.34, 3.05 | 0.89 | 0.70 | -2.57, 3.98 | 0.69 |
| TAG, % | 205 | -2.47 | -6.67, 1.92 | 0.27 | -3.25 | -7.04, 0.70 | 0.10 |
| Lp(a), % | 202 | 8.33 | -8.61, 28.4 | 0.35 | 5.44 | -11.40, 25.48 | 0.55 |
| OxLDL, % | 122 | -0.30 | -4.50, 4.08 | 0.90 | 0.60 | -3.63, 5.02 | 0.77 |
| **Metabolic markers** |  |  |  |  |  |  |  |
| Glucose, % | 201 | 0.60 | -0.90, 2.12 | 0.41 | -0.01 | -1.29, 1.31 | 0.99 |
| **Inflammatory markers** |  |  |  |  |  |  |  |
| CRP, % | 122 | -2.66 | -13.76, 9.86 | 0.66 | -2.86 | -13.67, 9.31 | 0.63 |
| RANTES, % | 122 | -1.98 | -3.92, 0.02 | 0.05 | -1.78 | -3.73, 0.30 | 0.09 |
| sCD40L, % | 122 | -0.60 | -10.15, 9.97 | 0.90 | 0.04 | -9.88, 10.96 | 0.99 |
| TNF, pg/mL | 122 | -0.07 | -0.16, 0.02 | 0.12 | -0.10 | -0.19, -0.01 | **0.03** |
| TGF-β, % | 122 | -2.76 | -8.70, 3.46 | 0.37 | -3.05 | -8.97, 3.15 | 0.32 |
| IFN-γ, % | 122 | -5.45 | -13.84, 3.77 | 0.24 | -6.29 | -14.62, 2.84 | 0.17 |
| IL-6, % | 122 | -4.11 | -11.40, 3.67 | 0.28 | -6.76 | -12.89, -0.10 | **0.05** |
| IL-8, % | 122 | -2.57 | -7.04, 2.12 | 0.28 | -4.21 | -8.42, 0.20 | 0.06 |
| IL-10, % | 122 | -2.57 | -10.68, 6.40 | 0.56 | -1.98 | -10.24, 7.04 | 0.65 |
| α_1_-antitrypsin, g/L | 205 | -0.02 | -0.04, 0.003 | **0.02** | -0.03 | -0.05, -0.01 | **0.02** |
| Lp-PLA2, % | 122 | 1.51 | -3.92, 7.25 | 0.59 | 2.12 | -3.44, 8.00 | 0.46 |
| Autotaxin, % | 122 | -1.00 | -3.82, 1.92 | 0.51 | -0.10 | -2.76, 2.63 | 0.94 |
| **Platelet markers** |  |  |  |  |  |  |  |
| Fibrinogen, g/L | 201 | 0.03 | -0.04, 0.09 | 0.45 | 0.003 | -0.06, 0.07 | 0.93 |
| PF4, pg/mL | 122 | -40.13 | -70.74, -9.51 | **0.01** | -41.41 | -72.54, -10.27 | **0.01** |
| NAP-2, pg/mL | 122 | -4.60 | -8.90, -0.30 | **0.04** | -4.74 | -9.17, -0.31 | **0.04** |
| **Fatty acids** |  |  |  |  |  |  |  |
| ALA, % of FAME | 122 | 0.02 | -0.01, 0.05 | 0.14 | 0.02 | -0.01, 0.05 | 0.15 |
| EPA, % | 122 | 15.14 | 8.33, 22.26 | **<0.001** | 12.64 | 6.50, 19.12 | **<0.001** |
| DHA, % of FAME | 122 | 0.21 | 0.13, 0.29 | **<0.001** | 0.18 | 0.11, 0.26 | **<0.001** |
| LA, % of FAME | 122 | 0.25 | -0.24, 0.75 | 0.32 | 0.52 | 0.10, 0.94 | **0.02** |
| AA, % of FAME | 122 | -0.06 | -0.28, 0.17 | 0.62 | -0.10 | -0.33, 0.12 | 0.36 |

The effect estimate (β coefficient) corresponds to the change in the outcome variable for every 1-point increase in dietary score, when all other predictors in the regression model are held constant.

CI, confidence interval; LDL-C, low-density lipoprotein cholesterol; HDL-C, high-density lipoprotein cholesterol; ApoA, apoliprotein A; ApoB, apolipoprotein B; TAG, triacylglycerol; Lp(a), lipoprotein(a); OxLDL, oxidized LDL; CRP, C-reactive protein; RANTES, Regulated upon Activation Normal T-cells Expressed and Secreted; sCD40L, soluble CD40 ligand; TNF, Tumor Necrosis Factor; TGF-β, Transforming Growth Factor β_1_; IFN-γ, Interferon γ; IL-6, Interleukin 6; IL-8, Interleukin 8; IL-10, Interleukin 10; Lp-PLA2, Lipoprotein-associated phospholipase A2; PF4, Platelet factor 4; NAP-2, Neutrophil-Activating Peptide-2; ALA, α-Linolenic Acid; FAME, fatty acid methyl ester; EPA, Eicosapentaenoic acid; DHA, Docosahexaenoic acid; LA, Linoleic acid, AA, Arachidonic acid.

^a^ *p*-values <0.05 are considered statistically significant (marked in bold). ^b^ Adjusted for gender, age, BMI, and smoking status.
